# Tyrosine Hydroxylase is crucial for maintaining pupal tanning and immunity in *Anopheles sinensis*

**DOI:** 10.1038/srep29835

**Published:** 2016-07-15

**Authors:** Liang Qiao, Minghui Du, Xin Liang, Youjin Hao, Xiu He, Fengling Si, Ting Mei, Bin Chen

**Affiliations:** 1 Institute of Entomology and Molecular Biology, College of Life Sciences, Chongqing Normal University, Chongqing, 401331, China

## Abstract

Tyrosine hydroxylase (TH), the initial enzyme in the melanin pathway, catalyzes tyrosine conversion into Dopa. Although expression and regulation of *TH* have been shown to affect cuticle pigmentation in insects, no direct functional studies to date have focused on the specific physiological processes involving the enzyme during mosquito development. In the current study, silencing of *AsTH* during the time period of continuous high expression in *Anopheles sinensis* pupae led to significant impairment of cuticle tanning and thickness, imposing a severe obstacle to eclosion in adults. Meanwhile, deficiency of melanin in interference individuals led to suppression of melanization, compared to control individuals. Consequently, the ability to defend exogenous microorganisms declined sharply. Accompanying down-regulation of the basal expression of five antimicrobial peptide genes resulted in further significant weakening of immunity. TH homologs as well as the composition of upstream transcription factor binding sites at the pupal stage are highly conserved in the *Anopheles* genus, implying that the *TH*-mediated functions are crucial in *Anopheles*. The collective evidence strongly suggests that *TH* is essential for *Anopheles* pupae tanning and immunity and provides a reference for further studies to validate the utility of the key genes involved in the melanization pathway in controlling mosquito development.

Cuticle tanning (pigmentation and sclerotization) and innate immunity are two physiological processes essential for survival and reproduction of insects. Cuticle tanning is associated with insect body plan integrity, maintaining normal molt development and protecting from exogenous physical injury, while innate immunity determines the ability to resist pathogens to maintain fitness in insects^1^,^2^,^3^,^4^. Previous researches on *Drosophila melanogaster* and other model insects have demonstrated that the melanin metabolism pathway is closely linked to these two fundamental physiological processes through provision of Dopa and other supporting metabolites^3^,^5^,^6^,^7^,^8^,^9^,^10^.

Adult mosquitoes feed on human blood, leading to adverse effects on health and development through transmission of a number of malignant infectious diseases (such as malaria, dengue, chikungunya, Zika fever and encephalitis). Almost one-third of the human population is under risk of mosquito-borne diseases that endanger millions of lives worldwide, therefore, mosquitoes present an essential type of disease vector and insect species of significant medical interest^11^,^12^. During mosquito development, larvae undergo pupal metamorphosis to molt into adults that directly threaten human health and lives. During the pupal metamorphosis process, cuticle tanning shapes and supports the body, reduces external physical damage, and increases heat absorption. In addition, melanin substrates generated during melanization are indispensible for cuticle tanning, that act to reinforce and thicken the cuticle, encapsulate and kill microbial pathogens, consequently protecting pupae from infection by pathogenic microorganisms in environmental water^3^,^13^,^14^,^15^,^16^,^17^. Therefore, normal melanization of pupae is crucial for adult mosquito survival, reproduction, disease spreading, and hemophagia.

Similar to other insects, production of melanin in mosquitoes is dependent on the specific melanin metabolism pathway. In this conserved pathway, Tyrosine hydroxylase (TH) catalyzes the initial substrate, tyrosine, into Dopa, an important melanin precursor^3^,^14^,^18^,^19^,^20^. More importantly, Dopa is crucial for cuticle tanning, melanotic encapsulation immune responses, and the production of other catecholamines (such as Dopamine, N-β-alanyldopamine (NBAD), N-acetyldopamine (NADA)) used to build the exoskeleton^3^,^18^,^19^,^20^. Therefore, the normal function and structural integrity of *TH* as the initial rate-limiting synthetic enzyme gene are vital for insect tanning and innate immunity. Previous researches on *Drosophila melanogaster*, *Bombyx mori*, *Tribolium castaneum* and other insects disclosed that dysfunctional *TH* results in abnormal development of cuticle or eggshell pigmentation and structure, clearly indicating its requirement in cuticle tanning. Moreover, studies on *Manduca sexta* supported the involvement of *TH* in the melanotic encapsulation response^21^,^22^,^23^,^24^,^25^,^26^. However, no reports to date have focused directly on the physiological function of the *TH* gene during pupal metamorphosis in mosquito.

In this study, the malarial vector, *Anopheles sinensis* (mainly distributed in Southeast Asia, Japan and Korea)^27^, was used as a model to explore the role of *Anopheles sinensis Tyrosine hydroxylase* (*AsTH*) during pupal development. Silencing the expression of *AsTH*, the fatal defective type, verified that *AsTH* is indispensable for cuticle pigmentation and sclerotization, subsequent eclosion and the ability to defend against exogenous microorganisms during pupal development. Based on the amino acid similarities of TH homologs as well as quantities and constitution of upstream transcription regulatory elements, we inferred that expression and regulation patterns, and consequently, functions of *TH* are conserved in *Anopheles*. Therefore, the requirement for the *TH* gene to maintain normal pupal development is likely to be ubiquitous in the genus *Anopheles*. Our findings aid in clarifying the characteristics and physiological roles of TH, and provide a platform to further investigate the utility of *TH* and other key genes involved in the melanin metabolism pathway in mosquito control strategies.

## Materials and Methods

### Mosquitoes

The *Anopheles sinensis* WX-LS strain was reared in the Institute of Entomology and Molecular Biology, Chongqing Normal University. Larvae were fed fry food in clean water at 27 °C under a 12 h: 12 h (light: dark) photoperiod until pupation. Eggs were gathered from 1 h to 1day after birth (pigmentation started from the first hour after oviposition). Adults were collected at 0, 3, 6 and 9 h after eclosion.

### Chemicals

Dopa for measurement of melanization was provided by Mr. Kunpeng Lu (State Key Laboratory of Silkworm Genome Biology, Southwest University, Chongqing, China).

### Identification of *Anopheles sinensis* TH

Tyrosine hydroxylase amino acid sequences of *Drosophila melanogaster* and *Anopheles gambiae* were used as the query sequences to search for homologous genes from the *Anopheles sinensis* genome, transcriptome database (www.vectorbase.org) and data from our laboratory (unpublished) through blastP and tblastN programs. The putative gene and its Expressed Sequence Tag sequences were assembled using the SeqMan program to obtain the longest *Anopheles sinensis TH* sequence.

### Cloning of *AsTH*

At 38 h of pupation, three pupae were ground in liquid nitrogen. Total RNA was isolated using TRizol reagent (Invitrogen, USA), and cDNA synthesized using the RT reagent kit (RR047A, Takara, Japan). Four pairs of primers were designed based on the putative *AsTH* fragment to obtain full-length *AsTH* gene. The 3′UTR of *AsTH* was amplified using the GeneRacer kit (Invitrogen, USA). PCR products were cloned into the pMD19-T vector for sequencing. The primers designed for cloning are listed in Table S1.

### Phylogenetic analysis

TH homologs were searched with blastP using NCBI (http://www.ncbi.nlm.nih.gov/), Vectorbase (http://www.vectorbase.org/), Bettlebase (http://beetlebase.org/), silkDB (http://www.silkdb.org/silkdb/) and MonarchBase (http://monarchbase.umassmed.edu/). Amino acid sequences were aligned using Muscle online program (http://www.ebi.ac.uk/Tools/msa/muscle/). Subsequently, the neighbor-joining method in MEGA5.0^28^ was used to construct the phylogenetic tree, and bootstrap values obtained based on 1000 bootstrap replications. The Jones-Taylor-Thornton (JTT) model was employed for computation^28^.

### Synteny and transcription factor binding site analyses in different insects

For the Synteny analysis, we used *Anopheles gambiae TH* and adjacent genes as the templates to search for corresponding homologs in other insect species, and identified their location and distribution using blastP and tblastN. Since the 5′ UTR region of *TH* is not integrated in some insects, we selected 6 kb upstream DNA sequences of *TH* 5′ UTR in insects (all insects experience metamorphosis development and the length of 5′ UTR ≥300 bp) to analyze the composition of the reported binding sites of the transcription factors responding to hormonal signals during pupal development. Transcription factor binding sites were predicted using JASPAR (http://jaspar.genereg.net/) online analysis program with 90% confidence settings.

### RT-PCR

Total RNA was extracted and purified from the whole body of *Anopheles sinensis* at several developmental stages (from egg to adult). RNA extraction was performed from tissues of pupae at 38 h of pupation, including integument and fat body, using TRizol reagent (Invitrogen, USA) according to the manufacturer’s protocol. cDNA was synthesized using the RT reagent Kit (Takara, Japan). Primers designed for RT-PCR are listed in Table S1. The *Ribosomal protein L49* (*RPL49*) gene was used as the internal control.

### Quantitative RT-PCR

Quantitative RT-PCR was performed to measure the expression levels of *Anopheles sinensis Dopa decarboxylase* (*AsDDC*) and *laccase2* (*Aslac2*) in dsRNA of *AsTH* (ds*TH*) and dsRNA of red fluorescent protein gene (ds*Red*) pupae (at 38 h of pupation) using the Bio-Rad CFX96 detection system (Bio-RAD, USA) with a SYBR Premix Ex-Taq kit (Bio-RAD, USA) according to the manufacturer’s protocol. The primers designed for qRT-PCR are listed in Table S1. *Ribosomal protein S7* (*RPS7*) was used as the internal control. Three biological replicates were examined per sample.

### RNAi of *AsTH*

We employed RNAi to validate the function of *AsTH*. The ds*TH*, ds*TH2* (used to verify off-target effects) and ds*Red* fragments were synthesized using the T7 RiboMAX^TM^ Express RNAi System (Promega, USA). The dsRNA was diluted to 6 μg/μL with RNase-free water, and a 800 ng dose was injected into each individual. The injection time was selected according to the temporal expression pattern of *AsTH* between 0 and 2 h after pupation. Subsequently, evaluation of RNAi effects via RT-PCR was performed in pupae at 38 h of pupation and adults at 6 h of eclosion. Additionally, expression of the antimicrobial peptide genes (*AMPs*), *Asattacin*, *Ascecropin-A*, *Ascecropin-B*, *Asdefensin* and *Asgambicin* were examined via RT-PCR in pupae at 38 h of pupation. Primers used for RNAi experiments and antimicrobial peptide genes are listed in Table S1. Data from statistical of RNAi experiments are presented in Table S2.

### Frozen Cuticle sections

Individuals with difficulties in eclosion and lighter cuticles, compared to the ds*Red* group, were selected from the ds*TH* group for dissection of dorsal plates. The ds*Red* individuals within the same period were used as the control group. Dorsal plates were digested for 30 min with proteinase K, washed with distilled water, dried, and subsequently embedded in the paper mold using embedding medium (Sakura, Japan). Consistent orientations and angles were maintained when placing dorsal plates. Freezing at −30 °C for 30 min was followed by serial sectioning with a Leica Frozen Section Machine (CM1900, Germany). Sections were observed under a microscope (OLYMPUS BX63, Japan), and the images were analyzed using Image J analysis application software. Frozen cuticle sections were obtained from four individuals in the ds*TH* and ds*Red* groups, respectively. Four measurements were performed for each individual within the same region of the dorsal plate (Fig. S3), and the averages calculated, representing cuticle thickness of the dorsal plate of each individual.

### Bacterial treatment

After RNAi injection, individuals were selected according to cuticle pigmentation status in the ds*TH* (those that failed to develop melanism) and control groups, respectively. Individuals at 26 h of pupation were injected with 0.12 μL *Serratia marcescens* (*Se*, gram-negative bacteria (G^−^)) and *Bacillus bombyseptie* (*Bb*, gram-positive bacteria (G^+^)) (OD_600_ = 0.6–0.8) cultured in Luria-Bertani medium. The survival rate was calculated every 2 h after injection.

### *In vivo* and *in vitro* melanization assays

Three pupae with the same-sized and significantly lighter cuticles in the ds*TH* group were ground at 4 °C with 180 μL PBS (pH = 7.0) and centrifuged (500 × *g* for 5 min at 4 °C). Pupae in the ds*Red* group during the same period were employed as the control. In total, 80 μL supernatant (protein concentration of 6 mg/mL quantified using the Bradford Method) was transferred to a PCR tube and incubated at 30 °C for 1 h, followed by the addition of 1 mM phenylthiourea (PTU) to terminate the reaction. Subsequently, *in vivo* melanization was examined, and A_490_ values measured to assay the amount of melanin. Meanwhile, 30 μL supernatant (protein concentration, 6 mg/mL) was homogenized with 90 μL of 1 mM Dopa (dissolved in distilled water with a small amount of HCl solution) and incubated at 30 °C for 1 h, followed by addition of 1 mM PTU for termination of the reaction, to observe *in vitr*o melanization. A_490_ values were measured to assess the amount of melanin. RT-PCR was performed to assay the expression levels of four pro-phenoloxidase (PPO) genes (*AsPPO2*, *AsPPO4*, *AsPPO5* and *AsPPO9*), with *RPL49* as the internal control. Primers designed for RT-PCR are listed in Table S1.

## Results

### Conservation of *TH* in homologs of the *Anopheles* genus

**Figure 1:**
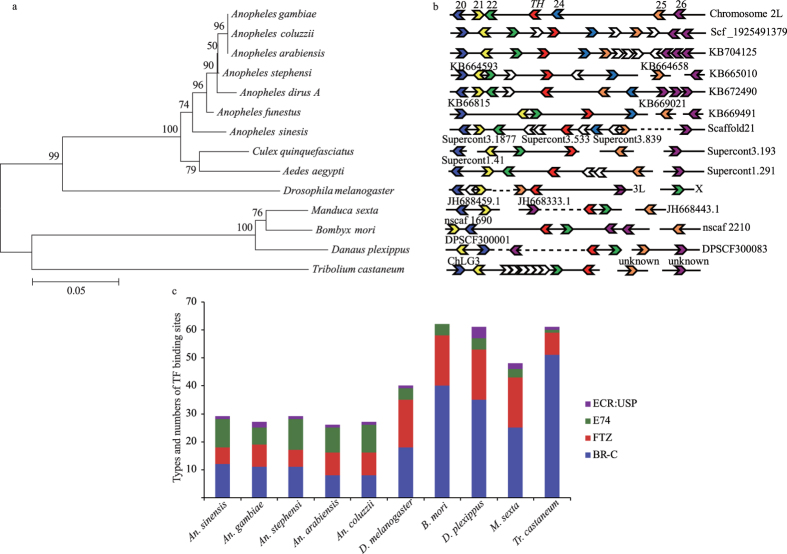
Characteristics of the *AsTH* gene. (**a**) Phylogenetic analysis of tyrosine hydroxylase (TH) among different insect species. Homologous amino acid sequences are listed as follows: *Anopheles Gambiae* gi|158295182|, *Anopheles coluzzii* ACOM024809, *Anopheles arabiensis* AARA007503, *Anopheles stephensi* ASTE005387, *Anopheles dirus A* ADIR008110, *Anopheles funestus* AFUN008134, *Culex quinquefasciatus* CPIJ014156, *Aedes aegypti* AAEL017098, *Drosophila melanogaster* gi|433470|, *Manduca sexta* gi|148611442|, *Bombyx mori* gi|223890158|, *Danaus plexippus* gi|357625078|, *Tribolium castaneum* gi|149588788|. A neighbor-joining tree of TH was generated using MEGA5.0^28^, with bootstrap values from 1000 replications. (**b**) synteny analysis of genomic regions containing the *TH* gene in different insect species. The order of synteny analysis corresponds to insect species in the phylogenetic tree. The genes within these genomic regions used to analyze synteny were adjacent to *TH* in *Anopheles Gambiae*. The horizontal solid line represents the chromosome or scaffold, and the other characters represent the chromosome or scaffold to which the genes belong. 20, 21, 22, *TH*, 24, 25, 26 and its own orthologs are represented by deep blue, yellow, green, red, light blue, orange and purple swallowtail symbols, respectively. The white swallowtail symbol represents the gene insert with no conserved orthologs among insect species. The direction of the swallowtail symbol represents that of gene transcription. The dotted line signifies insertion of multiple (more than 6) genes. (**c**) Prediction of representative transcription factor binding sites (ECR:USP, E74, FTZ, BR-C) involved in pupal gene expression within the 6 kb sequences upstream of *TH*.

The cloned *AsTH* gene (Genebank number: KU886220) was 4687 bp in length, with a 482 bp 5′ UTR and 2531 bp 3′ UTR (deduct poly(A)). The Biopterin-H domains of TH homologs in different insects are highly conserved (with identities ranging from 79 to 100%), particularly in the *Anopheles* genus (96 to 100% identity) (Fig. 1a, Fig. S1, Table S2). Moreover, the types and quantities of predicted genes adjacent to *TH* within the genomic region showed better synteny in the *Anopheles* genus, compared with other insect species (Fig. 1b). Remarkably, the predicted gene, *AGAP006024*, adjacent to *TH* in *Anopheles gambiae*, and its orthologs were solely identified in *Anopheles*, further supporting the theory that the *TH* genome is conserved within this genus (Fig. 1b). We further analyzed the reported representative transcription factors (ECR:USP, E74, FTZ, BR-C) that respond to hormones and regulate downstream gene expression in pupal development^29^,^30^ within the 6 kb upstream sequences of *TH*. Our data showed that the quantities and types of transcription factor binding sites in this region resemble each other to a higher extent in *Anopheles* than those in other insects (Fig. 1c), suggesting that the composition of regulatory elements in the *TH* upstream sequence is highly conserved in *Anopheles* genus. Consequently, transcriptional and regulatory processes of *TH* from these elements are more conserved, compared to those in other insects.

### The *AsTH* expression pattern is closely related to pupal cuticle tanning

**Figure 2:**
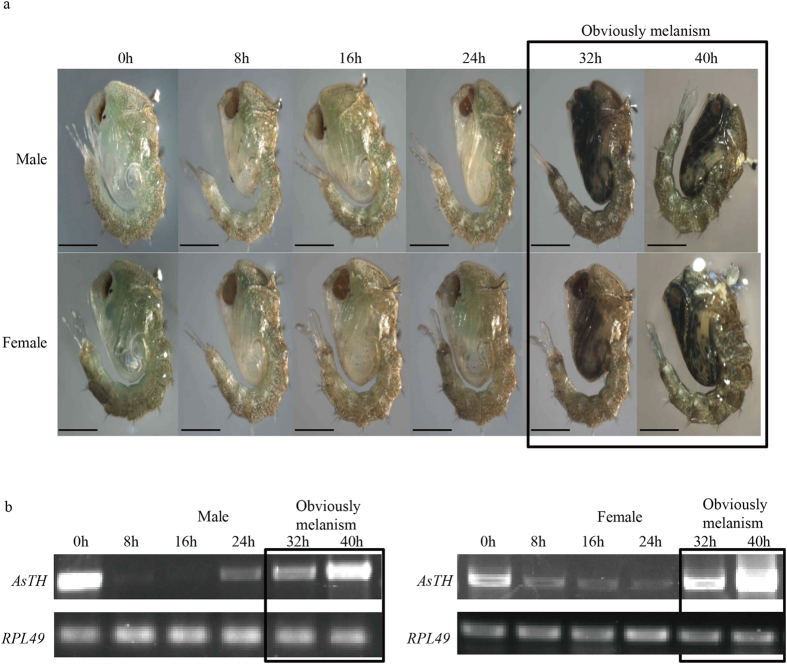
Relationship between *AsTH* expression patterns and phenotypic changes in cuticle tanning during *Anopheles sinensis* pupal development. (**a**) Phenotypic changes in male and female pupae at different stages of metamorphosis (from 0 to 40 h of pupation), and pupal cuticle melanism were distinct during 32 to 40 h (last phase) of pupation. (**b**) Temporal expression patterns of *AsTH* during pupal developmental stages in both male and female pupae. *RPL49* was used as the internal control.

Under the feeding conditions in our laboratory, *Anopheles sinensis* pupal metamorphosis in both males and females required about 42 h. During 0–24 h of pupal development, pupal melanism was not obvious, and only a small amount of yellowish pigment accumulated (Fig. 2a). After 24 h of pupation, pupae gradually became dark. In particular, from 32 h of pupation, distinct melanism was observed, particularly in the cephalothorax, and at 40 h, body color was the darkest before eclosion (Fig. 2a). Meanwhile, density and toughness of cuticle were significantly strengthened from 32 h to the last stage of pupation (40–42 h). Thus, during the mosquito pupal tanning process, key genes responsible for melanin formation should be very actively expressed at the late stages of pupal development. During pupal metamorphosis, *AsTH* expression was upregulated during the late stage of development, especially from 32 h of pupation (Fig. 2b). This expression trend of *AsTH* is consistent with pupal melanism. Therefore, based on the conservation of the *TH* sequence and its functions in insect cuticle pigmentation and sclerotization, we propose that *AsTH* is closely associated with *Anopheles sinensis* pupal tanning.

### Suppression of *AsTH* expression results in cuticle tanning defects

**Figure 3:**
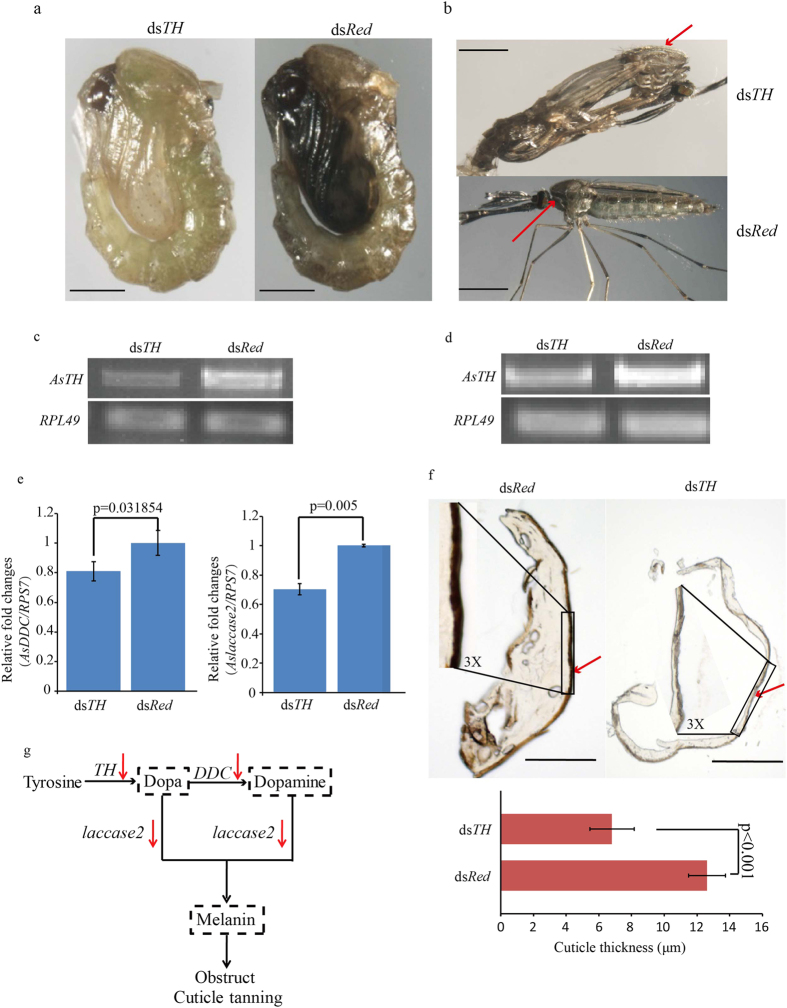
Effects of knockdown of *AsTH* during pupal developmental stages on cuticle tanning. (**a**) Pigmentation of pupae between the ds*TH* and ds*Red* groups at 38 h of pupation. Scale bar = 500 μm. (**b**) Eclosion and pigmentation states between ds*TH* and ds*Red* group adults at 6 h of eclosion. The red arrow indicates dorsal plates. Scale bar = 500 μm. (**c**) Expression levels of *AsTH* in ds*TH* and ds*Red* group pupae at 38 h of pupation. *RPL49* was used as the internal control. (**d**) Expression levels of *AsTH* in ds*TH* and ds*Red* adults at 6 h of eclosion. *RPL49* was used as the internal control. (**e**) Expression levels of *AsDDC* and *Aslaccase2* (downstream genes of *AsTH* in the melanin metabolism pathway) at 38 h of pupation. *RPS7* was used as the internal control (*t*-test, n = 3, *p* < 0.05 (for *AsDDC*), *p* < 0.01 (for *Aslaccase2*)). (**f**) Differences in color patterns and thickness of frozen sections of dorsal plates between ds*TH* and ds*Red* individuals. The red arrow represents across-section of the dorsal plate, and the black box represents the magnified area of the dorsal plate (scale bar = 200 μm). Magnification was described as X, *t*-test, n = 4, *p* < 0.001). (**g**) Schematic overview of cuticle tanning obstruction upon down regulation of *AsTH* expression. The red arrow represents suppression of gene expression, and the dotted frame signifies insufficiency of biochemical components.

By the 38^th^ h of pupation, the cuticle of normal pupa has already undergone melanism and sclerotization. The pigmentation trend of most pupae in the ds*TH* group began to slow down evidently from 26 h of pupation, compared to the ds*Red* group. Individuals in the ds*Red* group underwent distinct melanism, with significantly harder exoskeleton than when it was injected (0–2 h of pupation) (Fig. 3a). The developmental status of ds*Red* group individuals was not significantly different to that of normal pupae (with no treatment). However, the cuticles of individuals subjected to interference in the ds*TH* group failed to develop melanism until 38 h of pupation (only small shifts in color patterns, compared to neonatal pupae) and the exoskeleton was much softer than that of ds*Red* individuals (Fig. 3a). Moreover, at 6 h of eclosion, melanism was evident in the thorax of ds*Red* adults and exoskeleton sclerotization was complete (Fig. 3b). In contrast, thorax melanism of the ds*TH* individuals within the same period was not distinct, and individuals displayed a soft exoskeleton with lighter body color (Fig. 3b,f) and significantly thinner dorsal plate sections (~50%), compared to the *dsRed* individuals (Fig. 3f). Expression of *AsTH* was considerably lower than that of the control group at 38 h of pupation in pupae as well as adults (6 h of eclosion) (Fig. 3c,d). Expression of *AsDDC*^31^, the downstream gene of *AsTH*, was decreased by 20% at 38 h of pupation, compared to the control, due to knockdown of *AsTH* (Fig. 3e). Additionally, expression of *Aslaccase2*, an ortholog in other insects responsible for cuticle tanning^32^,^33^,^34^ was ~70%, compared to the control group individuals at 38 h of pupation due to catalytic substrate (Dopa-melanin and Dopamine-melanin) deficiency (Fig. 3e). In ds*TH* individuals, *AsTH* expression was significantly decreased, causing a cascade reaction leading to lower expression of other downstream key genes in the melanin metabolism pathway, and ultimately, pupal cuticle tanning impairment (Fig. 3g). To avoid off-target effects, we designed a ds*TH*2 fragment (that does not overlap with ds*TH*) for injection. Consequently, ~49% individuals failed to tan, and expression of *AsTH* was decreased (Fig. S2), verifying that the phenotype observed with *AsTH* gene RNAi is reliable. Defects in cuticle pigmentation and sclerotization present important phenotypic evidence to distinguish pupae with suppressed *AsTH* levels that provide a precise basis to select pupae for further immunity studies.

### Dysfunctional *AsTH* markedly impedes the eclosion process and rate

**Figure 4:**
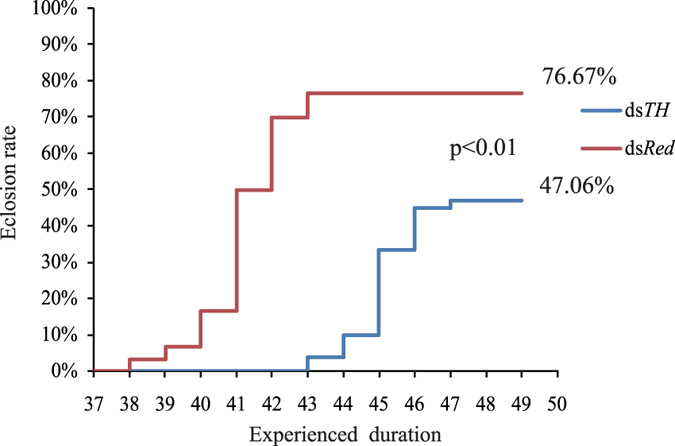
Comparison of eclosion rates and times between the ds*TH* and ds*Red* groups (log rank test, *p* < 0.01).

Individuals in the ds*TH* group failing to tan found it difficult to molt and eventually bled to death. Therefore, eclosion rate was decreased by ~30%, compared to the control group (77%, close to the natural eclosion rate of our laboratory strain) (Fig. 4). The remaining pupae that survived in the ds*TH* group required another 5 h to complete eclosion. However, these survivors were extremely feeble and found it difficult to fly and forage, ultimately leading to death as a result of *AsTH* dysfunction.

### Knockdown of *AsTH* significantly impairs *Anopheles sinensis* immunity

**Figure 5:**
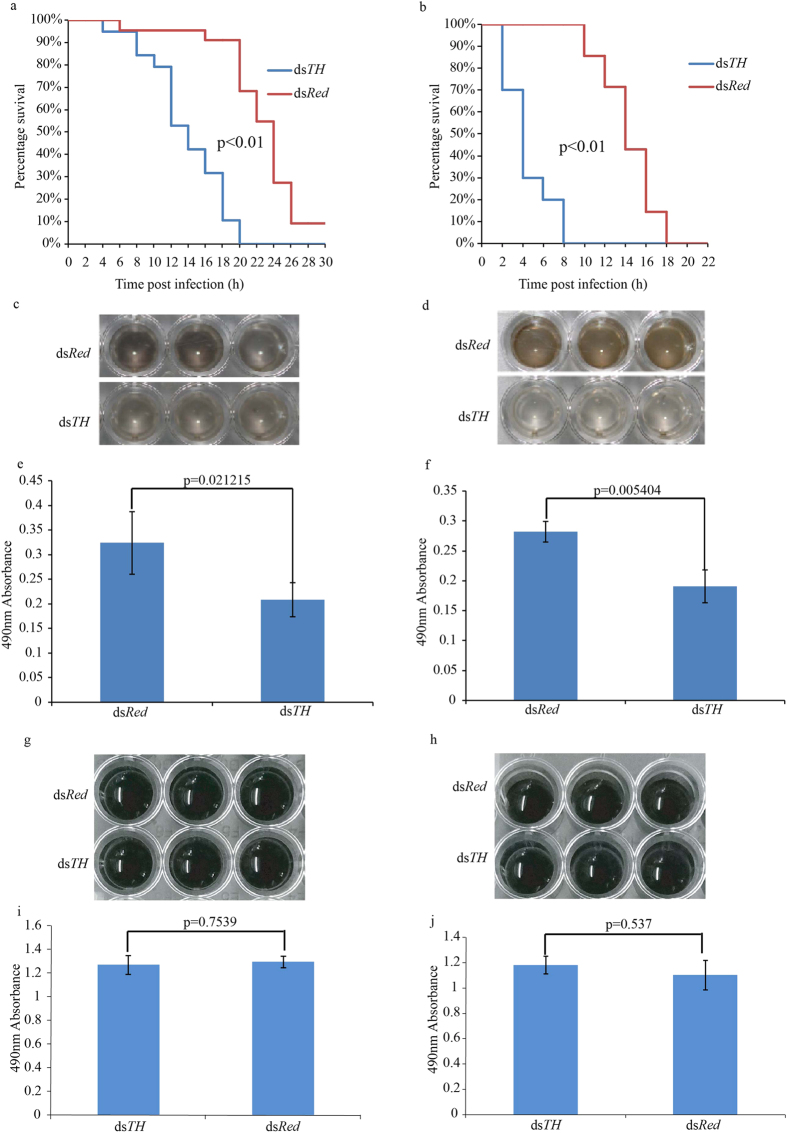
Effects of silencing the expression of *AsTH* on pupal resistance to exogenous microorganism infections. (**a**) and (**b**) indicate survival curves of ds*TH* and ds*Red* individuals subjected to *Bacillus bombyseptieus* and *Serratia marcescens* treatment, respectively (log rank test, *p* < 0.01). (**c**) and (**d**) present the degree of sample melanism of *in vivo* melanization after 1 h reaction. The degree of melanism in body fluid in ds*Red* group individuals is greater than that in ds*TH* individuals. (**e**) and (**f**) indicate the melanin amounts assessed from *in vivo* assay of ds*TH* and ds*Red* groups subjected to *Bacillus bombyseptieus* and *Serratia marcescens* treatment, respectively (*t*-test, n = 3, *p* < 0.01). (**g**) and (**h**) depict sample melanism of *in vitro* melanization after 1 h reaction. No obvious differences in the degree of melanism in body fluids between ds*Red* and ds*TH* groups are evident. (**i**) and (**j**) indicate melanin amounts assessed from *in vitro* assay of ds*TH* and ds*Red* individuals subjected to *Bacillus bombyseptieus* and *Serratia marcescens* treatment, respectively (*t*-test, n = 3, *p* > 0.5).

After knockdown of *AsTH*, survival rates and life spans of pupae in the ds*TH* group were dramatically decreased regardless of the type of bacteria used for treatment, compared to the ds*Red* group in the same experimental period (Fig. 5a,b). This finding provides direct evidence that the *AsTH* gene is indispensible for maintaining normal immunity in mosquitoes. Furthermore, the *in vivo* melanization assay showed that the degree of melanin production in the ds*TH* group was >30% lower than that in the control group after 1 h reaction regardless of the bacterial type injected (Fig. 5c–f). Our results indicate that melanin associated with melanotic encapsulation in the ds*TH* group is decreased during the innate immune response, leading to weakened immunity against exogenous microorganism infections. However, *in vitro* assays revealed no differences in the degree of melanization and amount of melanin after 1 h reaction between the ds*TH* and ds*Red* groups, regardless of the type of bacteria injected, as well as basal expression of *AsPPO* genes involved in melanization immunity^14^,^17^,^35^,^36^ (Fig. 5g–j, Fig. S6), suggesting that activities and amounts of phenol oxidases directly responsible for the melanotic encapsulation immune response do not differ significantly between the ds*Red* and ds*TH* groups.

## Discussion

The *TH* gene exhibits diverse temporal expression patterns at distinct developmental stages in different insect species (which may be closely related to the diversity of DNA regulatory elements in the upstream sequence), which are closely related to the physiological phenomenon and process in *TH* gene highly expression stages^21^,^22^,^23^,^24^,^25^,^26^,^37^,^38^,^39^,^40^,^41^,^42^. For example, in silkworm, high expression of *TH* at the late stage of embryonic development contributes to neonatal larvae mouthpart ossification via catecholamine (Dopa, Dopamine) synthesis^22^. Therefore, *TH* function determines whether the larvae can bite out of eggshells to completely hatch and subsequent foraging behavior. Data from our study suggest that expression levels of other genes in the melanin metabolic process are decreased after *TH* silencing that may lead to significant decrease in catecholamines (such as Dopa, Dopamine, and other precursors), resulting in a deficiency in the ingredients involved in cuticle pigmentation and sclerotization. Thus, pupal cuticle tanning is impaired, which ultimately causes serious physiological defects (Figs 3 and 4). The predominant expression period of *AsTH* in *Anopheles sinensis* pupae lasts longer than that in pupae of *Tribolium castaneum*^25^, consistent with *Anopheles sinensis* pupae experiencing more severe and extreme physiological defects upon *TH* silencing (dramatic decrease in eclosion rate and immunity), compared to *Tribolium castaneum* pupae. These data illustrate that *TH* is essential to maintain mosquito pupal development, particularly between the late pupal and early adult stages. The conservation of *TH* upstream regulatory elements in different *Anopheles* species further implies that *TH* gene expression patterns and functions are conserved in this genus (Fig. 1, Fig. S1), which is the key factor in mosquito-borne epidemics. Moreover, in terms of temporal expression patterns, we propose that high expression of *AsTH* in neonatal pupae (0 h of pupation) maybe related to cuticle sclerotization and yellowish pigment enhancement requiring a certain amount of catecholamines (such as NADA and NBAD) in early-middle pupae (ranging from 0 to 16 h of pupation) (Fig. 2). To our knowledge, this is the first study to report that dysfunction of *TH* induces phenotypic defects during mosquito pupa metamorphosis.

Our results are consistent with previous reports showing that *TH* is not only expressed in cuticle but also immune tissues, such as fat body, hemolymph and gut^23^,^26^,^43^ (Fig. S5). *TH* expressed in immune tissues may be involved in melanotic encapsulation against pathogenic microorganism infections^23^,^26^,^43^. However, direct phenotypic evidence is lacking, especially in mosquito pupa metamorphosis. In our experiments, *in vivo* melanotic encapsulation response was significantly decreased due to Dopa deficiency after *AsTH* silencing, consistent with the marked impairment of resistance to pathogenic microorganisms (Fig. 5a–f). Furthermore, in view of the similar amounts of substrate in the *in vitro* melanotic reaction, the phenol oxidase level was taken as the key factor determining the degree of melanosis. Since the melanin amounts in the *in vitro* melanotic reactions as well as basal expression of *PPOs* in both the d*sTH* and ds*Red* groups were similar (Fig. 5g–j, Fig. S6), the amount of phenoloxidase-catalyzed melanotic reaction does not appear to be affected by knockdown of *AsTH*, further verifying that melanotic degree differences *in vivo* between ds*TH* and ds*Red* groups are not related to phenol oxidases, and are only affected by the amount of Dopa. Accordingly, we speculate that the *TH* gene controls the source of melanotic encapsulation response through directly regulating the amount of endogenous Dopa (comparable with the regulatory pattern of phenylalanine hydroxylase in the upstream metabolic pathway)^44^ in live pupae.

**Figure 6:**
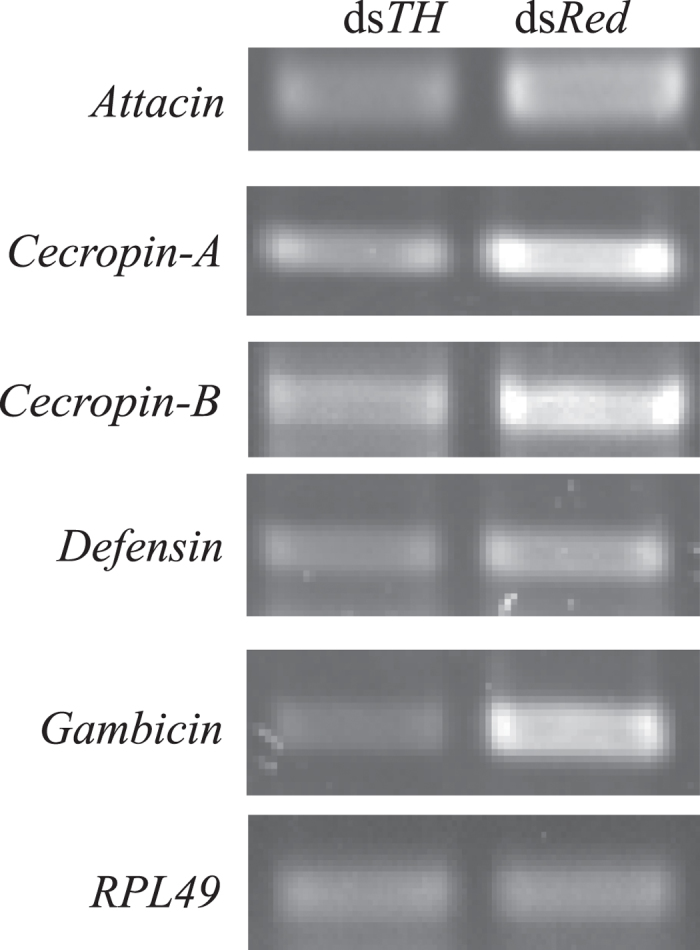
Basal expression levels of five antimicrobial peptide genes in ds*TH* and ds*Red* individuals at 38 h of pupation. *RPL49* was used as the internal control.

**Figure 7:**
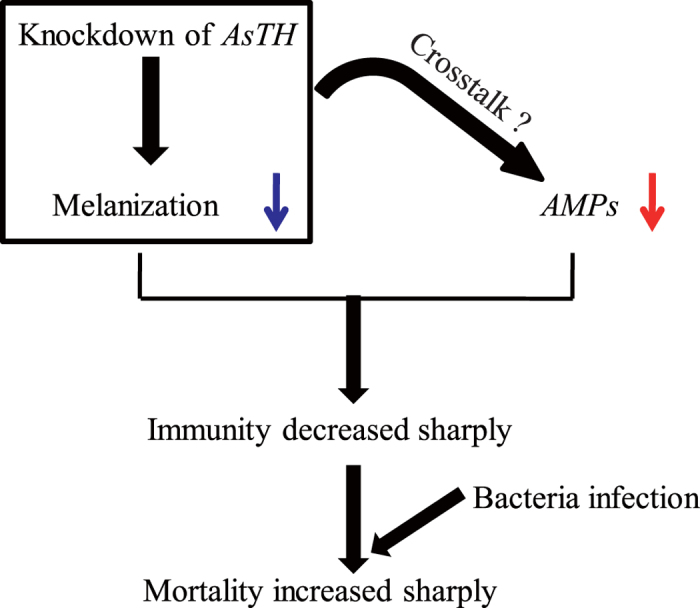
Schematic diagram of the pathways underlying decreased immunity upon knockdown of *AsTH* in *Anopheles sinensis* pupae. Blue and red arrows represent impaired melanization and basal expression of antimicrobial peptide genes, respectively.

Melanic body color in insects is usually accompanied by significantly enhanced immunity^5^,^16^,^24^,^45^. We propose that melanic body color stimulation by *TH* or other genes facilitating melanization leads to hardening and compacting of the cuticle to prevent pathogen invasion. A number of catecholamines (such as Dopa) in insect body are significantly involved in the melanotic encapsulation immune response to destroy pathogenic microorganisms. Our data showed decreased basal expression levels of some antimicrobial peptide genes, along with impaired cuticle melanism in the ds*TH* group (Fig. 6). This result is consistent with the previous finding that basal expression levels of *AMPs* in melanic *Galleria mellonella* are significantly higher, compared to their non-melanic counterparts^16^. Similarly, earlier research on silkworm showed that basal expression of *AMPs* in some melanic mutants are higher, compared to wild-type (non-melanic) individuals (unpublished data). Studies on *Drosophila melanogaster* and *Tribolium castaneum* revealed that melanization may affect *AMPs* expression^46^,^47^. We speculate that enhanced resistance to exogenous microorganisms is ascribed not only to melanotic immunity promotion but also activation of melanin production or the melanotic pathway through a specific route (perhaps crosstalk) inducing *AMPs* expression to enhance antimicrobial capacity, while conversely, insufficient melanin exerts an adverse effect on other immune pathways, resulting in a significant decline in immunity (Fig. 7). Further studies are required to explore this interesting phenomenon in *Anopheles sinensis*.

Silencing of *AsTH* during pupal metamorphosis led to serious physiological defects. Dysfunctional TH, resulting in cuticle failure to tan and inability to resist infection by microorganisms in water, would be fatal for pupae in the physiological environment. Given the irreplaceable role of *TH* in pupal metamorphosis, the gene may be utilized as the target with female or male pupae specifically expressing a genetic operating system to establish the CRISPER-Cas9 gene drive^48^,^49^,^50^ that can maintain the defective phenotype of dysfunctional *TH* stably and continuously in one-gender individuals, resulting in physiological defects or even death, while the other gender stably carries and inherits such genetic defects, to ultimately control the mosquito population. Our study provides a reference for potential utilization of the key genes in metabolic process indispensable for mosquito subsistence in developing effective control strategies.

## Additional Information

**How to cite this article**: Qiao, L. *et al*. Tyrosine Hydroxylase is crucial for maintaining pupal tanning and immunity in *Anopheles sinensis*. *Sci. Rep.* **6**, 29835; doi: 10.1038/srep29835 (2016).

## Supplementary Material

Supplementary Information

## References

[b1] Vincent J. & Hillerton J. The tanning of insect cuticle—a critical review and a revised mechanism. J Insect Physiol 25, 653–658 (1979).

[b2] Andersen S. O. Insect cuticular sclerotization: a review. Insect Biochem Mol Biol 40, 166–178 (2010). 19932179 10.1016/j.ibmb.2009.10.007

[b3] Hultmark D. Immune reactions in Drosophila and other insects: a model for innate immunity. Trends Genet 9, 178–183 (1993). 8337755 10.1016/0168-9525(93)90165-e

[b4] Schmid-Hempel P. Evolutionary ecology of insect immune defenses. Annu Rev Entomol 50, 529–551 (2005). 15471530 10.1146/annurev.ento.50.071803.130420

[b5] True J. R. Insect melanism: the molecules matter. Trends Ecol Evol 18, 640–647 (2003).

[b6] Sideri M., Tsakas S., Markoutsa E., Lampropoulou M. & Marmaras V. J. Innate immunity in insects: surface‐associated dopa decarboxylase‐dependent pathways regulate phagocytosis, nodulation and melanization in medfly haemocytes. Immunology 123, 528–537 (2008). 17983437 10.1111/j.1365-2567.2007.02722.x PMC2433321

[b7] Barnes A. I. & Siva-Jothy M. T. Density–dependent prophylaxis in the mealworm beetle Tenebrio molitor L.(Coleoptera: Tenebrionidae): cuticular melanization is an indicator of investment in immunity. Proc R Soc Lond B Biol Sci 267, 177–182 (2000). 10.1098/rspb.2000.0984 PMC1690519 10687824

[b8] Leger R. S., Cooper R. & Charnley A. The effect of melanization of Manduca sexta cuticle on growth and infection by Metarhizium anisopliae. J Invertebr Pathol 52, 459–470 (1988).

[b9] Kanost M. R., Jiang H. & Yu X. Q. Innate immune responses of a lepidopteran insect, Manduca sexta. Immunol Rev 198, 97–105 (2004). 15199957 10.1111/j.0105-2896.2004.0121.x

[b10] Kramer K. J. & Hopkins T. L. Tyrosine metabolism for insect cuticle tanning. Arch Insect Biochem Physiol 6, 279–301 (1987).

[b11] Caraballo H. & King K. Emergency department management of mosquito-borne illness: malaria, dengue, and West Nile virus. Emerg Med Pract 16, 1–23 (2014). 25207355

[b12] Tolle M. A. Mosquito-borne diseases. Curr Probl Pediatr Adolesc Health Care 39, 97–140 (2009). 19327647 10.1016/j.cppeds.2009.01.001

[b13] Johnson J. . A potential role for phenylalanine hydroxylase in mosquito immune responses. Insect Biochem Mol Biol 33, 345–354 (2003). 12609519 10.1016/s0965-1748(02)00257-6

[b14] Christensen B. M., Li J., Chen C.-C. & Nappi A. J. Melanization immune responses in mosquito vectors. Trends Parasitol 21, 192–199 (2005). 15780842 10.1016/j.pt.2005.02.007

[b15] Wilson K., Cotter S. C., Reeson A. F. & Pell J. K. Melanism and disease resistance in insects. Ecol Lett 4, 637–649 (2001).

[b16] Dubovskiy I. . More than a colour change: insect melanism, disease resistance and fecundity. Proc R Soc Lond B Biol Sci 280, 10.1098/rspb.2013.0584 (2013). PMC3774225 23698007

[b17] Osta M. A. Christophides G. K., Vlachou D. & Kafatos F. C. Innate immunity in the malaria vector Anopheles gambiae: comparative and functional genomics. J Exp Biol 207, 2551–2563 (2004). 15201288 10.1242/jeb.01066

[b18] Brunet P. Tyrosine metabolism in insects. Ann N Y Acad Sci 100, 1020–1034 (1963). 14016288 10.1111/j.1749-6632.1963.tb42948.x

[b19] Wittkopp P. J. & Beldade P. Development and evolution of insect pigmentation: Genetic mechanisms and the potential consequences of pleiotropy. Semin Cell Dev Biol 20, 65–71 (2009). 18977308 10.1016/j.semcdb.2008.10.002

[b20] Fuchs S., Rende E., Crisanti A. & Nolan T. Disruption of aminergic signalling reveals novel compounds with distinct inhibitory effects on mosquito reproduction, locomotor function and survival. Sci Rep 4, 5526, 10.1038/srep05526 (2014). 24984706 PMC4078307

[b21] Neckameyer W. S. & White K. Drosophila tyrosine hydroxylase is encoded by the pale locus. J Neurogenet 8, 189–199 (1993). 8100577 10.3109/01677069309083448

[b22] Liu C. . Repression of tyrosine hydroxylase is responsible for the sex-linked chocolate mutation of the silkworm, Bombyx mori. Proc Natl Acad Sci USA 107, 12980–12985 (2010). 20615980 10.1073/pnas.1001725107 PMC2919899

[b23] Lee K. S., Kim B. Y. & Jin B. R. Differential regulation of tyrosine hydroxylase in cuticular melanization and innate immunity in the silkworm Bombyx mori. J Asia Pac Entomol 18, 765–770 (2015).

[b24] Liu S., Wang M. & Li X. Overexpression of Tyrosine hydroxylase and Dopa decarboxylase associated with pupal melanization in Spodoptera exigua. Sci Rep 5, 11273, 10.1038/srep11273 (2015). 26084938 PMC4471665

[b25] Gorman M. J. & Arakane Y. Tyrosine hydroxylase is required for cuticle sclerotization and pigmentation in Tribolium castaneum. Insect Biochem Mol Biol 40, 267–273 (2010). 20080183 10.1016/j.ibmb.2010.01.004 PMC2854195

[b26] Gorman M. J., An C. & Kanost M. R. Characterization of tyrosine hydroxylase from Manduca sexta. Insect Biochem Mol Biol 37, 1327–1337 (2007). 17967351 10.1016/j.ibmb.2007.08.006 PMC2104791

[b27] Zomuanpuii R., Guruswami G. & Nachimuthu S. K. A three year study on distribution and ecology of Anophelines in Thenzawl, Mizoram, India. J Environ Biol 35, 369–376 (2014). 24665764

[b28] Tamura K. . MEGA5: molecular evolutionary genetics analysis using maximum likelihood, evolutionary distance, and maximum parsimony methods. Mol Biol Evol 28, 2731–2739 (2011). 21546353 10.1093/molbev/msr121 PMC3203626

[b29] Thummel C. S. Molecular mechanisms of developmental timing in C. elegans and Drosophila. Dev Cell 1, 453–465 (2001). 11703937 10.1016/s1534-5807(01)00060-0

[b30] Dubrovsky E. B. Hormonal cross talk in insect development. Trends Endocrinol Metab 16, 6–11 (2005). 15620543 10.1016/j.tem.2004.11.003

[b31] Huang C. Y., Chou S. Y., Bartholomay L., Christensen B. & Chen C. C. The use of gene silencing to study the role of dopa decarboxylase in mosquito melanization reactions. Insect Mol Biol 14, 237–244 (2005). 15926892 10.1111/j.1365-2583.2004.00552.x

[b32] Arakane Y., Muthukrishnan S., Beeman R. W., Kanost M. R. & Kramer K. J. Laccase 2 is the phenoloxidase gene required for beetle cuticle tanning. Proc Natl Acad Sci USA 102, 11337–11342 (2005). 16076951 10.1073/pnas.0504982102 PMC1183588

[b33] Elias-Neto M., Soares M. P., Simões Z. L., Hartfelder K. & Bitondi M. M. Developmental characterization, function and regulation of a Laccase2 encoding gene in the honey bee, Apis mellifera (Hymenoptera, Apinae). Insect Biochem Mol Biol 40, 241–251 (2010). 20184957 10.1016/j.ibmb.2010.02.004

[b34] Gorman M. J. . Kinetic properties of alternatively spliced isoforms of laccase-2 from Tribolium castaneum and Anopheles gambiae. Insect Biochem Mol Biol 42, 193–202 (2012). 22198355 10.1016/j.ibmb.2011.11.010 PMC3267840

[b35] Cerenius L. & Söderhäll K. The prophenoloxidase‐activating system in invertebrates. Immunol Rev 198, 116–126 (2004). 15199959 10.1111/j.0105-2896.2004.00116.x

[b36] Cerenius L., Lee B. L. & Söderhäll K. The proPO-system: pros and cons for its role in invertebrate immunity. Trends Immunol 29, 263–271 (2008). 18457993 10.1016/j.it.2008.02.009

[b37] Pendleton R. G., Rasheed A., Sardina T., Tully T. & Hillman R. Effects of tyrosine hydroxylase mutants on locomotor activity in Drosophila: a study in functional genomics. Behav Genet 32, 89–94 (2002). 12036114 10.1023/a:1015279221600

[b38] Futahashi R. & Fujiwara H. Melanin-synthesis enzymes coregulate stage-specific larval cuticular markings in the swallowtail butterfly, Papilio xuthus. Dev Genes Evol 215, 519–529 (2005). 16133568 10.1007/s00427-005-0014-y

[b39] Ninomiya Y., Tanaka K. & Hayakawa Y. Mechanisms of black and white stripe pattern formation in the cuticles of insect larvae. J Insect Physiol 52, 638–645 (2006). 16618489 10.1016/j.jinsphys.2006.03.002

[b40] Friggi‐Grelin F., Iche M. & Birman S. Tissue‐specific developmental requirements of Drosophila tyrosine hydroxylase isoforms. Genesis 35, 260–269 (2003). 12717737 10.1002/gene.1082

[b41] Liu J., Lemonds T. R. & Popadić A. The genetic control of aposematic black pigmentation in hemimetabolous insects: insights from Oncopeltus fasciatus. Evol Dev 16, 270–277 (2014). 25124093 10.1111/ede.12090 PMC4156130

[b42] Yu H.-S. . Evidence of Selection at Melanin Synthesis Pathway Loci during Silkworm Domestication. Mol Biol Evol 28, 1785–1799 (2011). 21212153 10.1093/molbev/msr002

[b43] Hashimoto K., Yamano Y. & Morishima I. Induction of tyrosine hydroxylase gene expression by bacteria in the fat body of eri-silkworm, Samia cynthia ricini. Comp Biochem Physiol B Biochem Mol Biol 149, 501–506 (2008). 18178496 10.1016/j.cbpb.2007.11.010

[b44] Fuchs S., Behrends V., Bundy J. G., Crisanti A. & Nolan T. Phenylalanine metabolism regulates reproduction and parasite melanization in the malaria mosquito. PLoS One 9, e84865 (2014). 24409310 10.1371/journal.pone.0084865 PMC3883676

[b45] Xiang Z., Huang J., Xia J. & Lu C. Biology of sericulture (ed. Xiang Z. ) Ch. 5, 119–130 (China For Publ House, Beijing, 2005).

[b46] Johnston P. R., Makarova O. & Rolff J. Inducible defenses stay up late: temporal patterns of immune gene expression in Tenebrio molitor. G3 (Bethesda) 4, 947–955 (2014). 10.1534/g3.113.008516 PMC4065263 24318927

[b47] Tang H. Regulation and function of the melanization reaction in Drosophila. Fly (Austin) 3, 105–111 (2009). 19164947 10.4161/fly.3.1.7747

[b48] Hammond A. . A CRISPR-Cas9 gene drive system targeting female reproduction in the malaria mosquito vector Anopheles gambiae. Nat Biotechnol 34, 78–83 (2016). 26641531 10.1038/nbt.3439 PMC4913862

[b49] Gantz V. M. . Highly efficient Cas9-mediated gene drive for population modification of the malaria vector mosquito Anopheles stephensi. Proc Natl Acad Sci USA 112, E6736–E6743 (2015). 26598698 10.1073/pnas.1521077112 PMC4679060

[b50] Adelman Z. N. & Tu Z. Control of mosquito-borne infectious diseases: sex and gene drive. Trends Parasitol 32, 219–229 (2016). 26897660 10.1016/j.pt.2015.12.003 PMC4767671

