# Supplementary material for: Tyrosine Hydroxylase is crucial for maintaining pupal tanning and immunity in Anopheles sinensis
Source: Sci Rep. 2016 Jul 15;6:29835. doi: 10.1038/srep29835 (PMC4945905; doi:10.1038/srep29835)
Supplement: Supplementary Information [file srep29835-s1.pdf]

Supplementary information

Tyrosine Hydroxylase is crucial for maintaining the pupae tanning and immune in *Anopheles sinensis*

Liang Qiao[1+*], Minghui Du[1+], Xin Liang[1], Youjin Hao[1], Xiu He[1], Fengling Si[1], Ting Mei[1], Bin Chen[1*]

[1]Institute of Entomology and Molecular Biology, College of Life Sciences, Chongqing Normal University, Chongqing, 400716, China.

[*]To whom correspondence should be addressed: Liang Qiao, Institute of Entomology and Molecular Biology, College of Life Sciences, Chongqing Normal University, Chongqing, 401331, China, Tel: +86-23-65910315, Fax: +86-23-65910315, E-mail address: qiaoliangswu@163.com, or Bin Chen, Institute of Entomology and Molecular Biology, College of Life Sciences, Chongqing Normal University, Chongqing, 401331, China, e-mail address: bin.chen@cqnu.edu.cn.

[+]Liang Qiao and Minghui Du contributed equally to this work.

Running title: *AsTH* for *Anopheles sinensis* pupae development

6 Supplemental Figures
3 Supplemental Tables

Supplementary Figure legends

Figure S1. Alignment analysis of TH amino acid sequences among different insect species. Horizontal line indicates the Biopterin-H domain.

Figure S2. Detection of RNAi off-target effects. a. The pupa cuticle pigmentation pattern at 38th hour of pupation between ds*TH2* and ds*Red* group. Scale bar=500μm. b. The expression level of *AsTH* between ds*TH2* and ds*Red* individuals at 38th hour of pupation. *RPL49* was used as the internal control.

Figure S3. Precise region of cuticle frozen section. The red box region represents the observed sliced section region. Scale bar=100μm.

Figure S4. The expression pattern of *AsTH* gene expression in the egg (eggs were gathered from 1 hour to 1 day after born) stage, larval stage and adult stage, respectively. *RPL49* was used as the internal control.

Figure S5. Spatial expression pattern of *AsTH* in different tissues at 38th hour of pupation. IN represents the integument, FB represents the fat body, (HE+G) represents the mixture of the hemolymph and the gut. The "*" symbol represents treatment with bacteria. *RPL49* was used as the internal control.

Figure S6. The expression levels of 4 representative *Anopheles sinensis* pro-phenoloxidase (PPO) genes (The orthologous genes in *Anopheles gambiae* were expressed in the immune tissues) between ds*TH* and ds*Red* group at 38th hour of pupation. *RPL49* was used as the internal control.

Figure S1

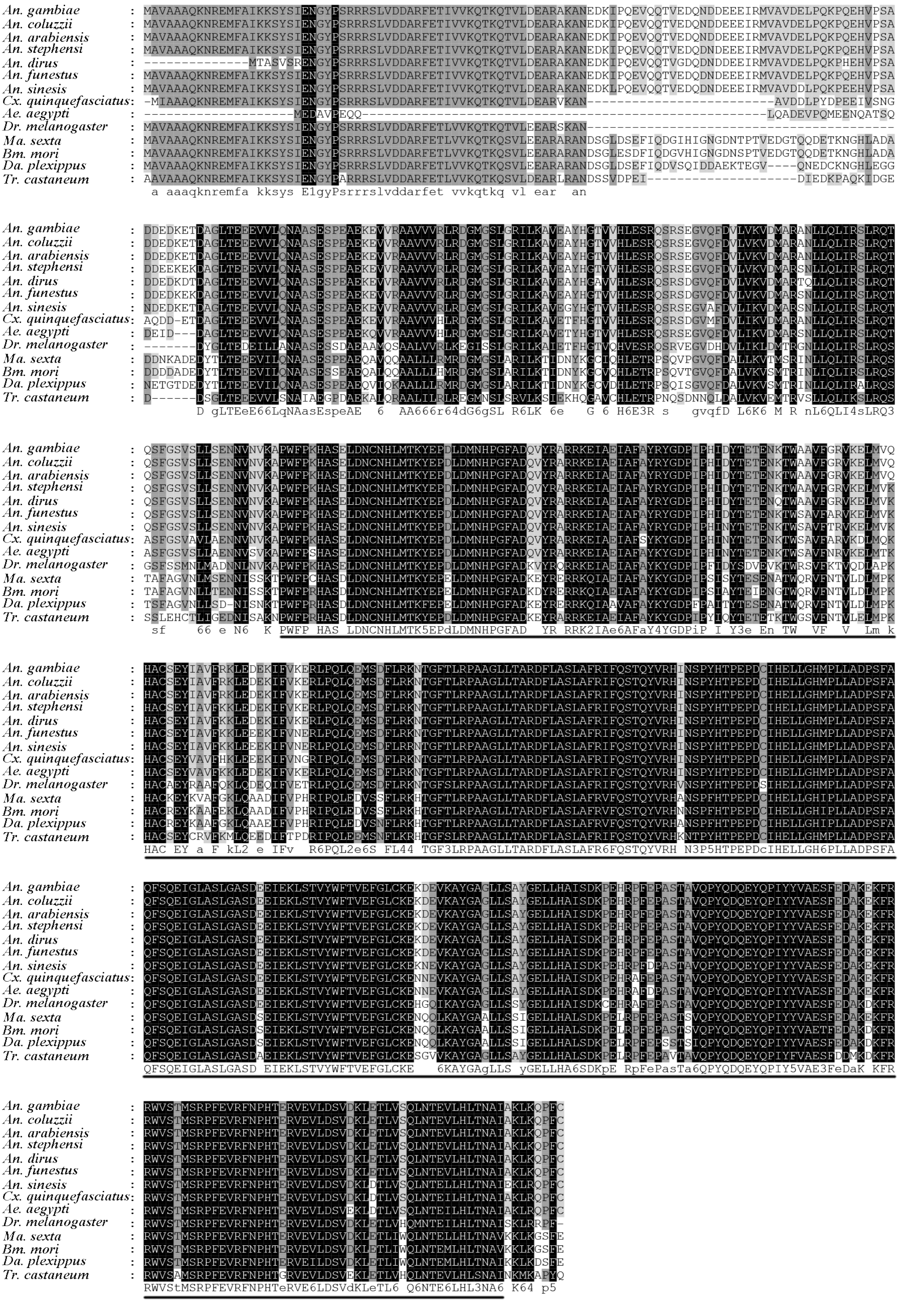
An. gambiae
An. coluzzii
An. arabiensis
An. stephensi
An. dirus
An. funestus
An. sinesis
Cx. quinquefasciatus
Ae. aegypti
Dr. melanogaster
Ma. sexta
Bm. mori
Da. plexippus
Tr. castaneum

Figure S2

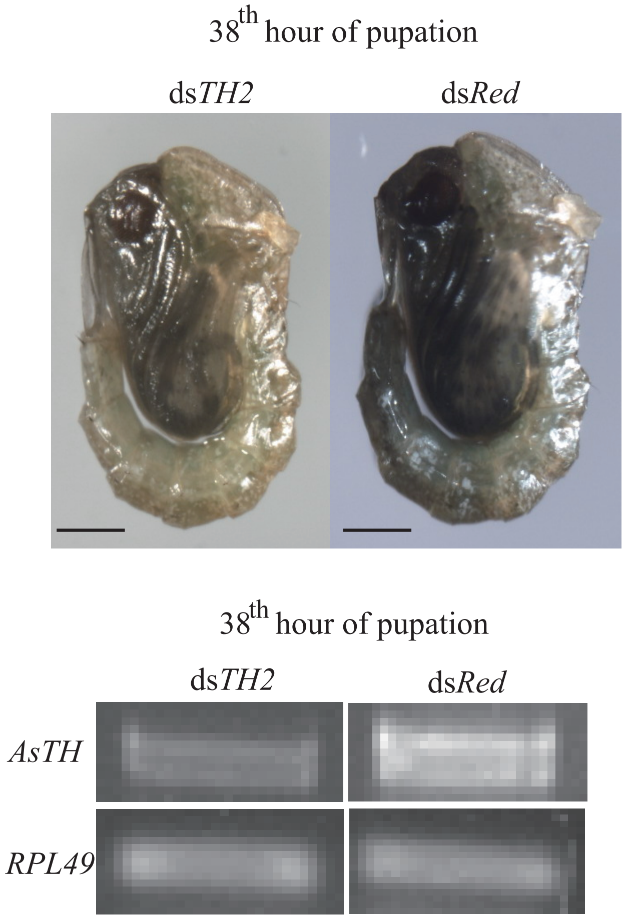
38th hour of pupation
dsTH2
dsRed
38th hour of pupation
dsTH2
dsRed
AsTH
RPL49

Figure S3

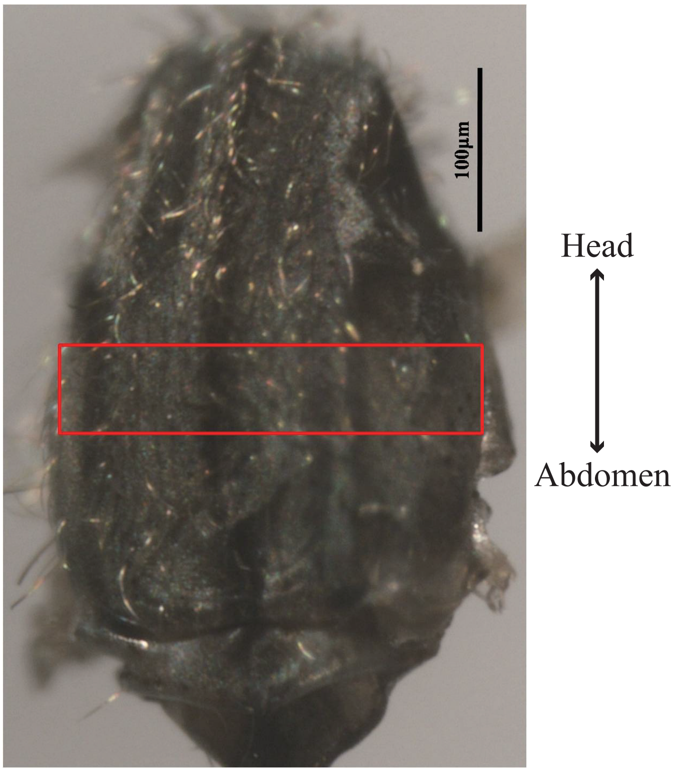

Figure S4

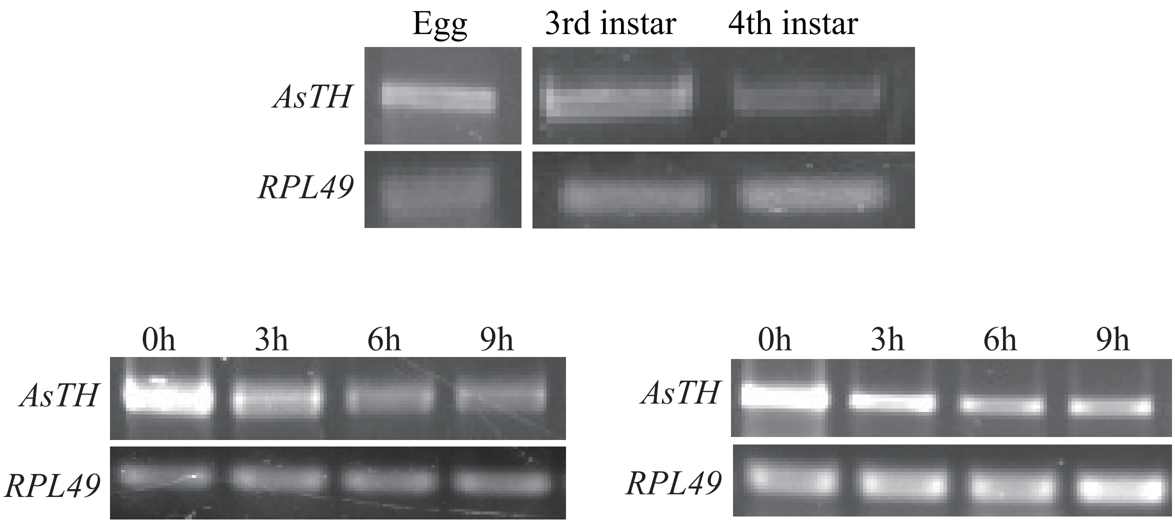
Egg
3rd instar
4th instar
AsTH
RPL49
0h
3h
6h
9h
AsTH
RPL49
0h
3h
6h
9h
AsTH
RPL49

Figure S5

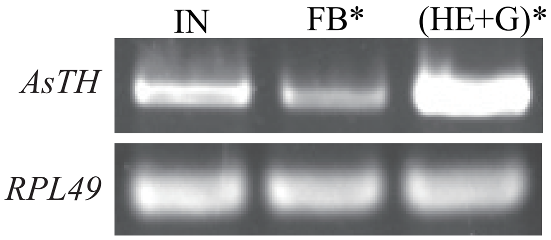

Figure S6

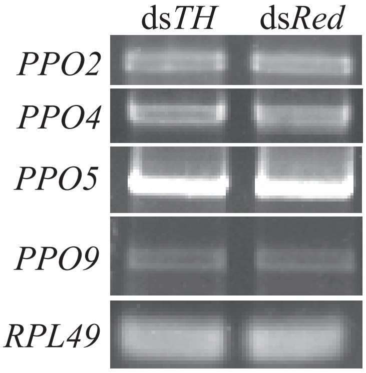

Table S1. Primers used in this study

Primer name	Sequence	
	F primer (5'---3')	R primer (5'---3')
Primers for *TH* cloning		
AsTH sequence 1	CCGATACCTGCCAAATCCAC	TGAGTCTGACGAGCCCTAACTCT
AsTH sequence 2	AAGACAAACTTCCGCAAGAGGT	CGTGTGGTAGGGCGAGTTG
AsTH sequence 3	CGGAAAGCGGTATGCATTCATA	TCATCCAGGACGGTTTGTTTG
AsTH sequence 4	CCAGCTCAACACCGAACTGC	AGGGGAAGAGGGAGGATGAAC
3'RACE nested primer	CAGAGTTAGGGCTCGTCAGACTCAT	CGCTACGTAACGGCATGACAGTG
3'RACE primer	CTTATGAGGACGGACAGAGTTAGGG	GCTGTCAACGATACGCTACGTAACG
Primers used in RT-PCR		
AsTH	GCTGGCGTTCCGCATCTT	GCGATGGCATTGGTGAGGT
RPL49	GGAGCCGGTCGGTGATATGT	TTCCTTCTCGGTCGGCTTCG
AsAttacin	CGGGTTTTTCCTGATTTGTGC	GCGACCGAAGTGCTGCGTAT
AsCecropin-A	ATCTTCGTATTCGTCCTGCTGG	GCCTTCTCTGCCGCCTTGA
AsCecropin-B	GCCTGTTCGTGGTGTTGATG	AGCAGTGCCTTCTGAGCCG
AsDefensin	ATCGTGCTGGTTGTGGTGGTA	GCAGGTTGCCCGCTTGAA
AsGambicin	TTCTCCTCGCCGTCCTCCT	CCGTATCGTTGGCAGTCCG
AsPPO 2	CTGGACGAGGAGCGTGGTAT	AGTCACATGGTGCGTTAGGATC
AsPPO 4	GTGCGATGGCGGTGTTTG	TCGTGGATGTAGCCGAGAATG
AsPPO 5	GTGCCTTCGCCGACATCA	CATTGTATCGCAAACGACCCT
AsPPO 9	CGGGTGTCACGCTATGGGA	TCTTCACGGCATCGTCTTCG
Quantitative RT-PCR primers		
AsDDC	GGACAGGGTGGTGGTGTTATTCA	GATTGCCTTCTCCAGCGTTTC
Aslaccase2	GTTCCCGTAACGAGCACACATT	CTCCAAACCGTCTTCGCATC
RPS7	CGGAGAAGATGGCATGGGAGAT	ATAGTGAGCATAGGCCCGGTTA
Primers used for dsRNA template amplification		
AsTH sequence 1	TAATACGACTCACTATAGGGAGAAAGACAAACTTCCGCAAGAGGT	TAATACGACTCACTATAGGGAGACGTGTGGTAGGGCGAGTTG
AsTH sequence 2	TAATACGACTCACTATAGGGAGACCGATACCTGCCAAATCCAC	TAATACGACTCACTATAGGGAGATGAGTCTGACGAGCCCTAACTCT
Red	TAATACGACTCACTATAGGGAGACTTCAAGGTGCGCATGGAG	TAATACGACTCACTATAGGGAGATGTGGATCTCGCCCTTCAG

Table S2. Amino acid identity of AsTH Biopterin_H domain with its orthologs

	An. gambiae	*An. sinesis*	*An. dirus*	*An. funestus*	*An. stephensi*	*An. arabiensis*	*An. coluzzii*	*Culex quinquefasciatus*	*Aedes aegypti*	*Drosophila melanogaster*	*Bombyx mori*	*Danaus plexippus*	*Manduca sexta*	*Tribolium castaneum*
An. gambiae	100%	96%	99%	98%	99%	100%	100%	93%	95%	87%	82%	81%	83%	84%
An. sinesis		100%	96%	97%	96%	96%	96%	95%	96%	86%	81%	80%	82%	85%
An. dirus			100%	98%	99%	99%	99%	93%	95%	87%	83%	81%	83%	85%
An. funestus				100%	98%	98%	98%	95%	95%	87%	83%	81%	83%	85%
An. stephensi					100%	99%	99%	93%	95%	88%	83%	81%	83%	85%
An. arabiensis						100%	100%	93%	95%	87%	82%	81%	83%	84%
An. coluzzii							100%	93%	95%	87%	82%	81%	83%	84%
Culex quinquefasciatus								100%	95%	85%	81%	79%	80%	83%
Aedes aegypti									100%	87%	82%	81%	83%	85%
Drosophila melanogaster										100%	83%	81%	83%	83%
Bombyx mori											100%	94%	96%	83%
Danaus plexippus												100%	94%	82%
Manduca sexta													100%	83%
Tribolium castaneum														100%

Table S3. Statistic of RNAi (For Cuticle tanning, at 38th hour of pupation)

Treatment	Injected No.	No. of individuals with tanning impaired (alive)	No. of Death at 38th hour of pupation
ds*TH*	58	32	6
ds*TH2*	42	18	5
dsRed (For ds*TH*)	49	0	5
dsRed (For ds*TH2*)	36	0	4
